# Transcriptional Regulatory Systems in Pseudomonas: A Comparative Analysis of Helix-Turn-Helix Domains and Two-Component Signal Transduction Networks

**DOI:** 10.3390/ijms26104677

**Published:** 2025-05-14

**Authors:** Zulema Udaondo, Kelsey Aguirre Schilder, Ana Rosa Márquez Blesa, Mireia Tena-Garitaonaindia, José Canto Mangana, Abdelali Daddaoua

**Affiliations:** 1 Department of Environmental Protection, Consejo Superior de Investigaciones Científicas, Estación Experimental del Zaidin, 18008 Granada, Spain; 2 Department of Biochemistry and Molecular Biology II, Pharmacy School, University of Granada, 18071 Granada, Spain; 3 Pharmacy Services, A.S. Hospital de Poniente de Almería, 04700 El Ejido, Spain; 4 Biosanitary Research Institute of Granada (IBS), 18014 Granada, Spain; 5 Institute of Nutrition and Food Technology “José Mataix”, Center of Biomedical Research, University of Granada, Avda. del Conocimiento s/n. Armilla, 18016 Granada, Spain

**Keywords:** gene expression, one-component system (OCS), two-component system (TCS), metabolism, *Pseudomonas putida* KT2440, *Pseudomonas aeruginosa* PAO1, pathogenicity

## Abstract

Bacterial communities in diverse environmental niches respond to various external stimuli for survival. A primary means of communication between bacterial cells involves one-component (OC) and two-component signal transduction systems (TCSs). These systems are key for sensing environmental changes and regulating bacterial physiology. TCSs, which are the more complex of the two, consist of a sensor histidine kinase for receiving an external input and a response regulator to convey changes in bacterial cell physiology. For numerous reasons, TCSs have emerged as significant targets for antibacterial drug design due to their role in regulating expression level, bacterial viability, growth, and virulence. Diverse studies have shown the molecular mechanisms by which TCSs regulate virulence and antibiotic resistance in pathogenic bacteria. In this study, we performed a thorough analysis of the data from multiple public databases to assemble a comprehensive catalog of the principal detection systems present in both the non-pathogenic *Pseudomonas putida* KT2440 and the pathogenic *Pseudomonas aeruginosa* PAO1 strains. Additionally, we conducted a sequence analysis of regulatory elements associated with transcriptional proteins. These were classified into regulatory families based on Helix-turn-Helix (HTH) protein domain information, a common structural motif for DNA-binding proteins. Moreover, we highlight the function of bacterial TCSs and their involvement in functions essential for bacterial survival and virulence. This comparison aims to identify novel targets that can be exploited for the development of advanced biotherapeutic strategies, potentially leading to new treatments for bacterial infections.

## 1. Introduction

Bacteria are highly adaptable organisms, capable of quickly adjusting to environmental changes by regulating their metabolism and gene expression [1]. To this end, they have evolved several features, including signal transduction systems that enable intracellular regulation and facilitate crosstalk between the intracellular and extracellular environments. However, this same adaptability has also contributed to the recent emergence of bacterial strains with multidrug resistance (MDR) phenotypes, which has become a serious health problem worldwide, constituting one of the main causes of human mortality [2].

*Pseudomonas* is a Gram-negative bacterial genus commonly found in soil, water, and plants, with species distributed globally [3]. They are widespread in the environment due to their exceptional adaptive capacity [4]. While some *Pseudomonas* species are generally harmless, others can cause infections in humans, animals, and plants [5], especially, in patients with compromised immune systems or those with medical devices, like catheters or prosthetic joints. *Pseudomonas* infections can range from mild skin to severe respiratory infections due to the genus’s antibiotic resistance [6,7]. On the other hand, certain strains within this genus have been successfully used in bioremediation control and industrial applications, showcasing their ability to degrade a wide range of organic compounds [8,9,10,11]. For example, *Pseudomonas putida* KT2440 is known for its beneficial effects on plants, due to its plant growth-promoting capabilities and its ability to efficiently colonize plant roots [12,13]. In contrast, other species, such as *Pseudomonas aeruginosa,* are versatile and opportunistic nosocomial pathogens that can infect both animals and plants [3,14].

**Figure 1:**
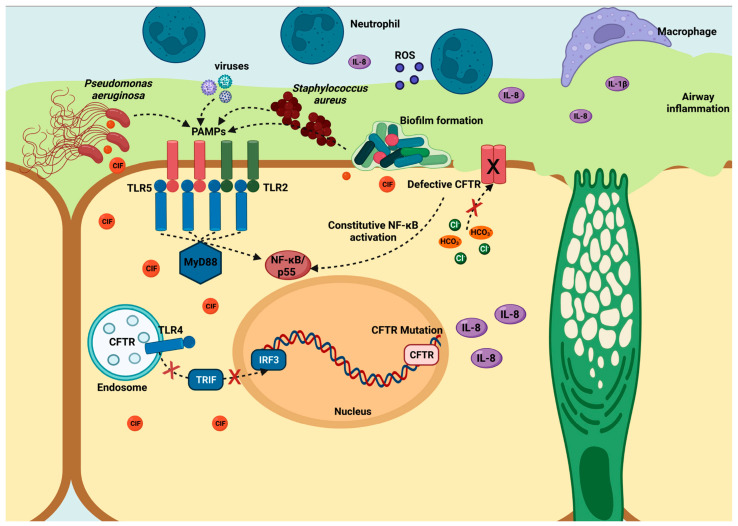
Airway epithelial adaptation of pathogens in cystic fibrosis and chronic obstructive pulmonary disease. TLRs’ activation (TLR2, TLR4, and TLR5) triggers MyD88-dependent and -independent responses. The MyD88-dependent pathway involves TIRAP and leads to NF-κB activation, resulting in cytokine production (IL-8, TNF-α). In contrast, the MyD88-independent pathway, mediated by TRIF, activates IRF3 and also contributes to immune responses. Defective CFTR in cystic fibrosis disrupts ion transport, leading to constitutive NF-κB activation, chronic inflammation, and cytokine production, contributing to disease pathogenesis.

*Pseudomonas aeruginosa* is a common cause of hospital-acquired infections, including ventilator-associated pneumonia [15] and catheter infections, particularly in immunocompromised patients and those suffering from cystic fibrosis (CF) [16] and severe burns [17,18]. Cystic fibrosis is a genetic disorder caused by mutations in the cystic fibrosis transmembrane conductance regulator gene (CFTR), which plays a crucial role in epithelial cell function and the regulation of epithelial fluid transport in the airways and other organs. The defective CFTR impairs normal airway function and disrupts the epithelium’s ability to interact effectively with pathogens. This dysfunction activates Toll-like receptors (TLRs), triggering downstream signaling via the MyD88-dependent pathway and subsequent activation of the NF-κB pathway, leading to the production of inflammatory cytokines, such as IL-6 and IL-8. This inflammatory response, illustrated in Figure 1, is a critical component of the body’s defense against bacterial infections, including those caused by *P. aeruginosa*, which frequently colonizes the lungs of cystic fibrosis patients. In addition to its direct effects on airway epithelial cells, CFTR dysfunction also contributes to systemic issues, such as intestinal barrier dysfunction [19,20], and alterations in the intestinal microbiota (dysbiosis). These changes are exacerbated by factors such as prolonged antibiotic use, obesity, diabetes, and fatty liver, promoting *P. aeruginosa* colonization in the intestinal ecosystem [21,22]. The persistence of *P. aeruginosa* infections in cystic fibrosis patients can be attributed to the bacterium’s adaptive capabilities acquired through mutation [23,24] and horizontal gene transfer [24,25], as well as its proficiency in biofilm formation [26,27,28] and the production of virulence factors, like Exotoxin A (ToxA) [29,30]. ToxA, found in over 90% of clinical isolates, disrupts protein synthesis by ADP ribosylation of elongation factor 2 (EF2), leading to cell death [31,32,33,34]. Furthermore, *P. aeruginosa* utilizes various secretion systems to deliver virulence factors either extracellularly or directly into host cells, enhancing bacterial survival and replication in the hostile environment of cystic fibrosis-affected tissues. These secretion systems range from type I to type VI secretion systems (T1SS to T6SS), enabling the bacterium to evade immune detection and maintain chronic infections in cystic fibrosis patients [35,36,37].

Nowadays, most antibiotics are discovered through screening processes designed to identify substances that inhibit bacterial growth [38]. However, these processes typically target a limited range of cellular activities. Bacteria develop resistance primarily through efflux pumps, which expel antibiotics and enzymes, such as beta-lactamases, which modify the drugs [39]. Additionally, strains like *P. aeruginosa* PAO1 and *P. putida* KT2440 have evolved complex signal transduction systems that detect and respond to environmental signals, enabling them to alter their cellular makeup in response to changing conditions.

These systems allow the bacteria to sense external stimuli, including the presence of signaling molecules in the environment. Upon detection, the systems efficiently relay these signals into the cell, initiating a cellular response that allows the bacteria to modify their cellular composition appropriately.

**Figure 2:**
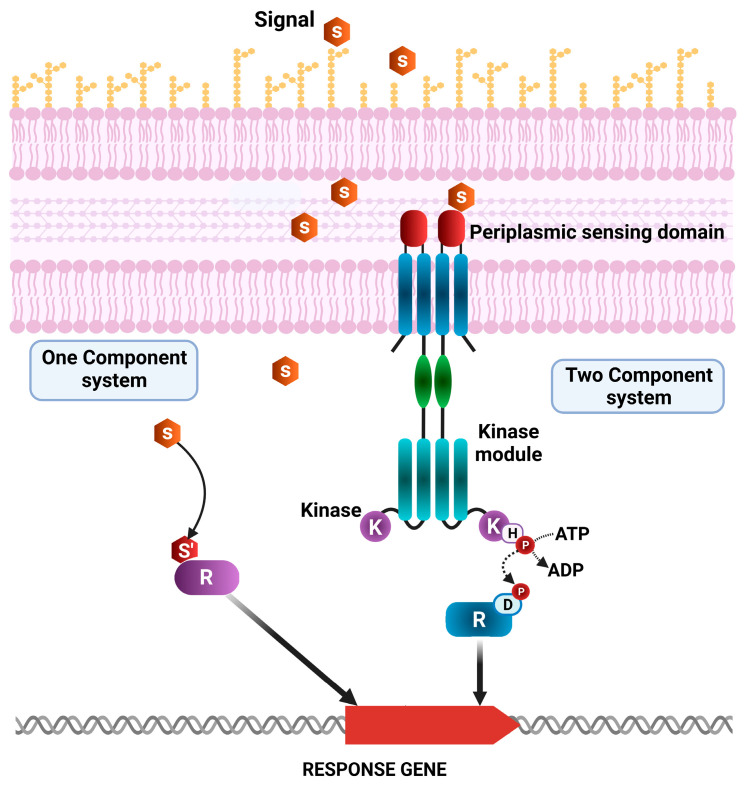
Schematic representation of one-component system (OCS) and two-component system (TCS) classes of *P. putida* KT2440 and *P. aeruginosa* PAO1 transcriptional regulators. R: Response regulatory protein; E: ligand-binding domain; S: signal molecules; K: histidine kinase, involved in phosphorylation/dephosphorylation events that activate the response regulator.

A prior analysis encompassing over 1000 genomes of diverse bacterial species revealed that signal transduction in bacteria is primarily mediated by proteins from two major superfamilies: one-component regulatory systems (OCSs) and two-component regulatory systems (TCSs) (Figure 2) [40,41,42,43,44,45,46]. In the context of TCSs, a transcriptional regulator generally consists of a histidine kinase (HK) protein or sensor, and a cognate response regulatory protein (R), which together modulate diverse signal transduction pathways (Figure 2). Sensor HKs possess multiple domains, including a periplasmic sensor domain for recognizing specific signals, a signal transduction domain, a cytoplasmic sensor domain, an adenosine triphosphate (ATP) catalytic domain, and a dimerization histidine phosphotransfer domain (D). The distribution of sensor proteins has been shown to vary, even among closely related microorganisms [43].

Upon the detection of a stimulus through the periplasmic or cytoplasmic detection domains, the HK sensor initiates the autophosphorylation of the conserved histidine residue within the HK domain. The phosphate group is then transferred to the aspartate residue of the R protein, which modulates the expression of genes involved in cellular responses. The effector domain of the R protein undergoes a conformational change upon phosphorylation, enabling it to bind or release DNA, thereby initiating changes in gene expression [44,45,46,47,48,49]. The responses elicited by these signal transduction systems can be highly diverse. In addition to regulating gene expression, certain response domains also mediate interactions with RNA, small ligands, other proteins, or exhibit enzymatic or transporter activities. However, one of the greatest challenges in understanding the complex bacterial signal transduction networks lies in identifying the signaling molecules that bind to sensor domains, initiating the signaling cascade [50].

In contrast, OCSs consist of proteins encompassing both a sensory and a DNA-binding domain but lacking the histidine kinase domains and extracellular receptors (Figure 2). Therefore, in these systems, a single protein performs both the detection of the intracellular signal and the initiation of the cellular response. Despite their simpler structure, these systems are more ancient and broadly distributed than TCSs. Both types of systems display a similar range of input and output domains. Hence, besides their structural differences, they may be able to detect similar stimuli and activate comparable cellular responses.

In this study, we analyzed the data from various public databases to compile an updated and detailed catalog of the main detection systems found in both the non-pathogenic *P. putida* KT2440 and the pathogenic *P. aeruginosa* PAO1 strains. Our primary goal was to delineate the significant distinctions in the protein components of signal transduction systems between these pathogenic and non-pathogenic bacteria. This comparison aims to elucidate the biological mechanisms that underpin the development of pathogenic traits.

Furthermore, we identified and cataloged several transcriptional regulators exclusive to the genome of *P. aeruginosa*, highlighting their pivotal roles in promoting bacterial virulence. This paper explores the unique attributes of these regulators and their potential as targets for therapeutic intervention. By linking the genomic characteristics of these transcriptional regulators to pathogenic traits, our research provides valuable insights for the development of novel therapeutic strategies.

## 2. Results and Discussion

### 2.1. Understanding the Genomic Sequences of P. aeruginosa PAO1 and P. putida KT2440: An Overview

**Figure 3:**
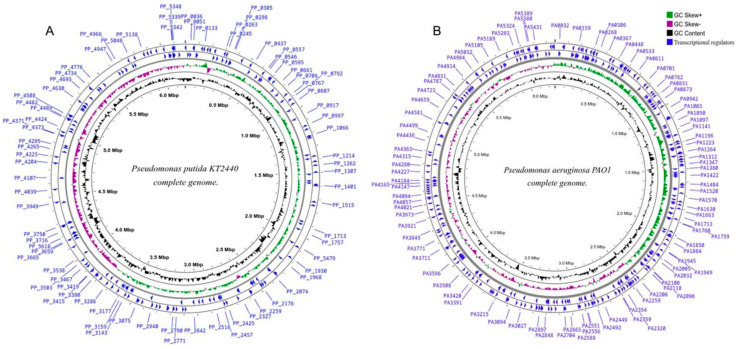
Circular genome analysis of (**A**) *Pseudomonas putida* KT2440 and (**B**) *Pseudomonas aeruginosa* PAO1 using the CGView tool (http://genocat.tools/tools/cgview_server.html) (Version not specified). The blue circus ring represents the distribution of genes identified as transcriptional regulatory.

The strain *P. putida* KT2440 (GCA_000007565.2) has a genome size of 6.2 Mbp with 5693 predicted genes (Figure 3A) [51,52], and *P. aeruginosa* PAO1 (GCA_000006765.1) boasts a genome spanning over 6.3 Mbp housing a predicted set of 5697 genes (Figure 3B) [53] Notably, *P. aeruginosa* PAO1 showcases a predicted array of 381 proteins falling under the transcription regulator classification, from which 44 identified as extra-cytoplasmic function (ECF) sigma factors [54]. The transcriptional activity has great relevance in this organism because many TCSs that respond to environmental conditions confer upon this bacterium the status of an infectious agent. The complex regulatory interplays within *P. aeruginosa* can be effectively visualized in the form of a transcriptional regulatory network. 

### 2.2. The Set of DNA-Binding Transcriptional Regulators in the Non-Pathogenic P. putida KT2440 and Pathogenic P. aeruginosa PAO1 Strains

**Figure 4:**
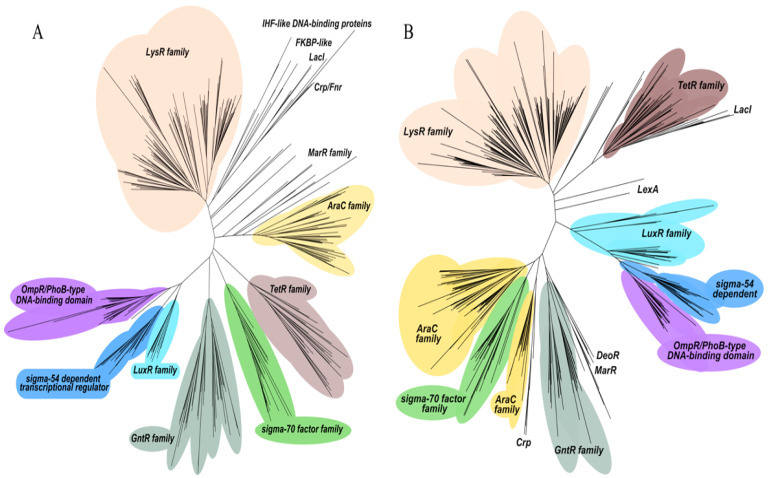
*Pseudomonas putida* KT2440 (**A**) and *Pseudomonas aeruginosa* PAO1 (**B**) phylogenetic tree representation of transcriptional regulators of the Helix-turn-Helix family. The colored area corresponds to sequences with an HTH family domain.

Understanding and linking gene expression to regulatory and physiological properties in bacterial strains becomes more achievable by identifying and characterizing the set of DNA-binding transcriptional regulators. Therefore, the genome sequences of both the environmental strain *P. putida* KT2440 and the opportunistic pathogen *P. aeruginosa* PAO1 were examined to detect genes encoding transcriptional regulators. Our data collection analysis revealed a total of 319 and 381 genes from transcriptional regulators (Figure 4) in the genomes of *P. putida* KT2440 and *P. aeruginosa* PAO1 (Tables S1 and S2), respectively.

Notably, a total of 27 and 44 genes of *P. putida* KT2440 and *P. aeruginosa* PAO1, respectively, were annotated as sigma factors. However, only 285 genes in *P. putida* KT2440 (Figure 4A and Table S1) and 331 genes in *P. aeruginosa* PAO1 (Figure 4B and Table S2) were classified within the HTH family (Helix-Turn-Helix). Interestingly, while the strain KT2440 contains 44 genes associated with TCSs, strain PAO1 harbors 81 genes associated with these systems.

### 2.3. Categorization of Transcriptional Regulators in P. putida KT2440 and P. aeruginosa PAO1 into Regulatory Protein Families

**Figure 5:**
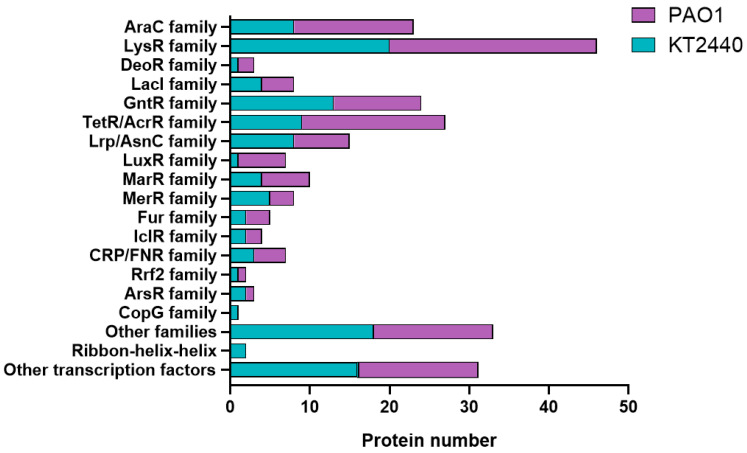
Categorization of DNA-binding transcriptional regulators in *P. putida* KT2440 and *P. aeruginosa* PAO1 into distinct regulatory protein families. The names of the identified regulatory protein families are shown. These families are labeled based on designations from the Pfam database.

Transcriptional regulators can be grouped into evolutionary regulatory protein families based on their amino acid sequence similarities [55]. Using amino acid sequence alignments obtained from the KEGG database, we categorized the complete collection of transcriptional regulators in *P. putida* and *P. aeruginosa* into 16 HTH-characterized protein families (Table S3). This included 120 regulators from KT2440, 142 from PAO1, and 18 from another unclassified HTH family (Figure 5 and Table S3). The majority of the identified regulatory protein families show a uniform size distribution among their assigned members, indicating a high degree of amino acid sequence similarity and suggesting a shared evolutionary origin. The number of identified regulatory protein families varies significantly, with the LysR family being the largest family with up to 20 and 26 members in *P. putida* KT2440 and *P. aeruginosa* PAO1, respectively (Table S3). In contrast, some protein families, like GntR, DeoR, LuxR, MerR, FuR, IcIR, CRp/FNR, RrF2, CopR, and ArsR, have as few as one-to-three members (Figure 5).

The LysR and GntR families of transcriptional regulators are extensively found in prokaryotes, including *P. putida* and *P. aeruginosa* strains. These families share a conserved structure comprising two functional domains: a conserved N-terminal DNA-binding Helix-Turn-Helix (HTH) motif and a C-terminal coinducer-binding or oligomerization domain. Typically, they exhibit negative autoregulation and are involved in the activation of a single divergently transcribed gene, influencing various cellular processes, like cell motility, glucose metabolism, bacterial resistance, pathogenesis, and virulence [56,57]. Significant distinctions in the transcriptional regulatory repertoire between pathogenic *P. aeruginosa* and non-pathogenic *P. putida* KT2440 are evident when comparing the number of proteins categorized into the TetR/AcrR, AraC, and LuxR families (Figure 5 and Table S3). The TetR/AcrR protein families are significantly prevalent in both *Pseudomonas* bacterial genomes. These families constitute a large group of OCS proteins that play a crucial role in regulating various processes, including efflux regulation, cell division, and stress responses processes critical for antimicrobial resistance [58,59].

Additionally, TetR/AcrR belongs to a transcriptional regulator family made up of two-domain proteins: an N-terminal HTH DNA-binding motif and a C-terminal ligand recognition domain. This design enables this regulator to recognize chemical sensors, to monitor various aspects of cellular environmental dynamics, such as antibiotics production, carbohydrate metabolism, osmotic stress, efflux pumps function, multidrug resistance, cellular virulence, and biofilm formation [58,60]. This diversity in DNA-binding transcriptional regulators certainly makes sense, given their role as chemical sensors or responding to environmental fluctuations. The prevalence of proteins in the GntR family may provide these bacteria the ability to thrive on various carbon sources and promptly adapt their gene expression in response to environmental shifts. Furthermore, this might indicate that pathogenic bacteria require a less versatile carbohydrate metabolism, as their natural habitats provide limited access to a diverse range of carbon sources.

It is noteworthy that alterations in the number of TetR/AcrR chemical regulatory sensors might correlate to variations in environmental conditions. For instance, pathogenic bacteria often tend to import the compound directly from the cell instead of relying on external sources. In particular, the number of regulatory proteins belonging to the TetR family is notably higher in the pathogenic species (Figure 5 and Table S3). This finding suggests that the TetR repressor family can serve as a universal switch for governing gene expression in *P. aeruginosa*, crucial for its more complex lifestyle and the need to regulate a wider array of genes associated with pathogenicity and resistance.

### 2.4. A Differential Repertoire Transcriptional Regulator Protein Is Evident in Pathogenic and Non-Pathogenic Pseudomonas Strains

As evidenced in Table S4, both pathogenic and non-pathogenic strains exhibit a similar number of genes encoding regulatory proteins, with 103 in KT2440 and 106 genes in PAO1. Additionally, our analysis identified a collection of 69 TCSs transcriptional regulators genes in KT2440, while the chromosomal sequences of PAO1 revealed 90 TCSs transcriptional regulator genes (Table 1). These findings suggest that the presence of TCSs is generally linked to a greater complexity in the regulation of gene expression, a phenomenon likely shaped by bacterial evolution.

### 2.5. Two-Component System Function in Pathogenic and Non-Pathogenic Pseudomonas Strains

**Figure 6:**
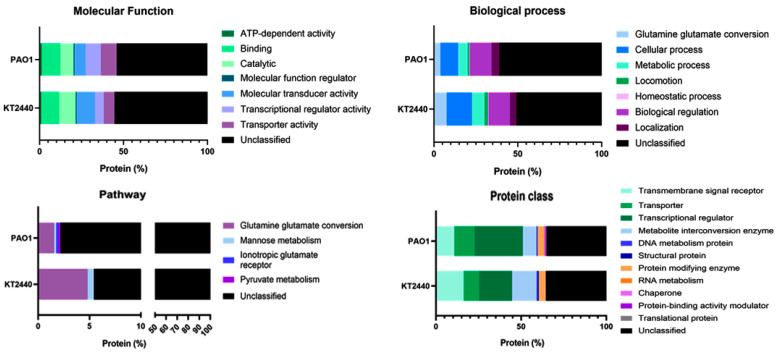
Functional comparison of genes from TCSs in *P. putida* KT2440 and *P. aeruginosa* PAO1. Functions are categorized into four groups: molecular function, biological process, pathway, and protein class. The primary functional Panther-GO categories are represented as a percentage of transcriptional regulators compared to the total number of HTH regulatory genes.

The ability of bacterial strains to respond to external stimuli is mediated by a specialized signal transduction mechanism, which relies on TCSs [47,48]. When activated, the sensor protein within the TCS catalyzes the autophosphorylation of a conserved histidine residue using adenosine triphosphate (ATP). The phosphoryl group is then transferred to a conserved aspartate residue in the regulatory protein (R), which may alter its ability to bind DNA sequences [45,47]. In recent years, a number of techniques have been developed to study two-component signal transduction systems [61], enabling the identification of stimuli-responsive TCSs. It has also been reported that some TCSs regulate gene clusters that contribute to cell growth, biofilm formation, and virulence in pathogenic bacteria [50,62,63,64]. However, in several cases, the role of TCSs in bacterial pathogenicity remains poorly understood. For instance, while TCS mutant strains often display attenuated virulence, the precise mechanisms underlying this effect are not yet fully elucidated. Functional analyses are essential for definitively determining the role of these sensors in the early stages of the infection process. Our findings suggest that these mechanisms and associated genes could serve as indicators of the diverse behaviors exhibited by different strains (Table S5 of KT2440 and Table S6 of PAO1). The results presented offer insights into the categorization and distribution of TCS transcriptional regulators in *P. putida* KT2440 and *P. aeruginosa* (Figure 6) based on PANTHER classifications.

Molecular Function: The comparative analysis of the molecular functions between *P. putida* KT2440 (Table S5) and *P. aeruginosa* PAO1 (Table S6) strains reveals both similarities and intriguing differences (Figure 6). In both strains, there were relatively few genes associated with “Transcription Regulator Activity”; 5.24% and 8.93% in KT2440 and PAO1, suggesting that specific genes play a role in modulating transcriptional processes. A significant proportion of genes in both strains is associated with “Binding Activity”, which facilitates molecular interactions and binding with other molecules. In KT2440 and PAO1 (Figure 6), 10.47% and 11.17% of genes, respectively, are involved in “Binding Activity”. Furthermore, “Catalytic Activity” accounts for 9.95% of genes in KT2440, compared to 7.90% in PAO1, indicating similar functional requirements in both strains. Notably, genes in *P. aeruginosa* PAO1 linked to “Molecular Transducer Activity” (6.70%) and “Transporter Activity” (9.45%) displayed a distinct profile compared to *P. putida* KT2440 (10.99% and 6.28%, respectively). Interestingly, only a few genes were involved in “ATP-Dependent Activity” (1.05% in KT240 and 1.20% in PAO1), implying a limited dependence on ATP for certain molecular functions. It should be noted that, in the strain KT2440, a significant proportion of genes (55.50%) compared to (54.12%) in PAO1 remain “Non-Characterized”, indicating a need for further investigation to shed light on the functional significance and potential applications of these unique molecular functions in these strains.

Biological process: Surprisingly, the total number of genes involved in biological processes differs significantly between non-pathogenic *P. putida* KT2440 and *P. aeruginosa* PAO1. In the KT2440 strain, this number stands at 212 genes, while in *P. aeruginosa* it is three-times higher (594 genes), indicating a more complex transcriptional regulation system (Figure 6 and Table S6). Specifically, the “Cellular Process” category accounts for 15.09% in KT2440 and 10.61% in PAO1 of the TCSs and encompasses fundamental cellular activities. These regulators are likely pivotal in controlling essential cell functions. Additionally, involvement in cellular localization processes is marked by 3.77% in KT2440 and 4.55% in PAO1, suggesting that a subset of transcriptional regulators plays a role determining the location of cellular components, which could be crucial for cell organization and structure. However, a notable proportion of these regulators, 50.94% in KT2440 and 61.11% in PAO1 (Figure 6), fall into no specific category. This lack of categorization highlights a substantial gap in our understanding of the roles and classifications of these transcriptional regulators. Consequently, further research and more detailed categorization are crucial for gaining a comprehensive understanding of their functions (Figure 6).

Pathway: The comparative analysis using PANTHER Pathway distributions (Figure 6) between *P. putida* KT2440 (Table S5) and *P. aeruginosa* PAO1 (Table S6) reveals notable differences in the types and proportions of genes associated with specific pathways. In this analysis, 166 genes were identified in the KT2440 strain and 512 in PAO1. A significant majority of these genes, 94.58% in KT2440 and 97.85% in PAO1, were categorized as “NO PANTHER category assigned” suggesting a considerable gap in our current understanding of the functional categorization of these genes. Specific pathways, such as “Glutamine glutamate conversion” and “Mannose metabolism”, were represented by less than 3.27% and 0.4% of the genes in KT2440 and PAO1, respectively. In contrast, both the “Ionotropic glutamate receptor” and “Pyruvate metabolism” pathway showed an exclusive representation in PAO1 at 0.20%, compared to KT2440 (Table S5 of KT2440 and Table S6 of PAO1). These slight variations in pathway representation may reflect distinct biological characteristics and functional preferences unique to each bacterial strain.

Protein class: In *P. putida* KT2440, genes categorized under “Transmembrane Signal Receptor” represent a significant 16.27% result, suggesting their vital role in cellular signaling processes. “Transporter” genes, accounting for 9.04%, are integral in the transportation of molecules across cellular membranes. Genes identified as “Gene-Specific Transcriptional Regulator” (19.28%) play a pivotal role in regulating gene expression and transcriptional processes. “Metabolite Interconversion Enzyme” genes, making up to 14.46%, underscore the importance of metabolic transformations in this strain. However, the mere 0.6% of “DNA Metabolism Protein” genes suggests a minor role in DNA metabolism. “Structural Protein” genes (0.6%) hint at their involvement in cellular structure, while “ProteinModifying Enzyme” genes (4.22%) are likely involved in modifying the structure or function of other proteins. A substantial portion of genes (35.54%) remains “Unclassified”, highlighting a gap in our understanding of their functions (Figure 6, Table S5 of KT2440 and Table S6 of PAO1).

Contrastingly, in *P. aeruginosa* PAO1, the “Transmembrane Signal Receptor” genes comprise 10.81%, indicating a significant but reduced role in cellular signaling compared to *P. putida* KT2440. “Transporter” genes at 11.98% are crucial for molecular transportation across cellular compartments. The substantial presence of “Gene-Specific Transcriptional Regulation” genes (28.49%) points to an active involvement in gene regulation processes. “Metabolite Interconversion Enzyme” genes (8.06%) parallel the role seen in KT2440 (Figure 6). The 0.39% of “DNA Metabolism Protein” genes suggests a less prominent role in DNA metabolism than in *P. putida* KT2440. “Structural Protein” genes (0.2%) and “Protein Modifying Enzyme” genes (3.73%) have roles in structural support and protein modification, respectively. “Protein Binding Activity Modulator” genes (0.39%) are involved in modulating protein-binding activities. However, a significant 35.36% of genes remain “Unclassified”, calling for further investigation of these genes.

In summary, compared to *P. putida* KT2440, *P. aeruginosa* PAO1 exhibits a similar percentage (16.27%) of genes in the “NO Panther Category” and a higher percentage in the “Gene-specific transcriptional regulation” and “Transporter” classes (Figure 6, Table S5 of KT2440 and Table S6 of PAO1) compared to KT2440. Conversely, *P. putida* KT2440 has a greater proportion of genes related to “Transmembrane signal receptor” and “Metabolite interconversion enzyme”. These variations highlight the distinct genetic composition and functional capacities inherent to these strains.

### 2.6. Forecasting and Choice of Transcriptional Regulators Associated with Pathogenicity

The exploration of regulatory mechanisms controlling the expression of virulence factors in bacterial pathogens reveals promising avenues for therapeutic intervention. In this context, two-component systems (TCSs) have emerged as compelling targets for the development of novel antibacterial agents [63]. Unlike traditional antibiotics that typically inhibit essential bacterial proteins, targeting TCSs offers a strategy to disrupt upstream regulatory networks, thereby impairing a pathogen’s ability to adapt and express virulence determinants. For example, in *P. aeruginosa* PAO1, TCSs such as PhoPQ, GacSA, and PmrAB regulate a suite of virulence-associated genes, including *toxA* (exotoxin A), *exoS*, *exoT*, and the components of the type III secretion system, like pscC and popB. These regulators also influence secondary metabolite production, including pyocyanin and rhamnolipids.

By contrast, *P. putida* KT2440 harbors TCSs, such as GacSA and FleSR, which are mainly involved in environmental sensing, motility, and type VI secretion system (T6SS) regulation, but it lacks several key effectors associated with pathogenicity, reflecting its reduced virulence potential [65,66]. The absence of genes like *toxA* and *exoU* further underscores its classification as a non-pathogenic, environmentally adapted species.

These genomic distinctions align with their divergent ecological roles: *P. aeruginosa* is a recognized opportunistic pathogen capable of forming biofilms, evading host immunity, and thriving in polymicrobial settings (Table 2), whereas *P. putida* is widely regarded as a chassis for industrial and biotechnological applications. As indicated in Table 2, genes are grouped according to the system involved, such as motility, secretion systems, immune modulation, biofilm, quorum sensing, metabolites, virulence factors, and others.

Because TCSs regulate antibiotic resistance determinants, such as mexXY (controlled by ParRS protein) and arnBCADTEF (regulated by PhoPQ and PmrAB proteins) in *P. aeruginosa* [67], therapeutic strategies combining TCS inhibitors with conventional antibiotics could enhance efficacy and mitigate resistance development.

Importantly, the reliance of bacterial TCSs on histidine phosphorylation, a mechanism absent in mammals, suggests that specific inhibitors would have minimal off-target toxicity. Moreover, the conserved architecture of histidine kinase and response regulator domains raises the possibility of designing broad-spectrum inhibitors capable of targeting multiple TCSs simultaneously, thus reducing the likelihood of chromosomal resistance emergence.

## 3. Material and Methods

### 3.1. Genomic Analysis

The general method used to identify DNA-binding transcriptional regulators in sequenced *Pseudomonas* genomes involved exploring combination of diverse bioinformatics databases. Putative DNA-binding proteins were initially searched for in the complete genome sequences of *Pseudomonas aeruginosa* PAO1 (RefSeq: GCA_000006765.1) and *Pseudomonas putida* KT2440 (RefSeq: GCF_000007565.2) using keywords, sequence similarity techniques, and also the databases PROSITE-Expasy (Swiss Institute of Bioinformatics, Lausanne, Switzerland; https://prosite.expasy.org/) (accessed on 6 December 2024), KEGG (Kyoto Encyclopedia of Genes and Genomes, Kyoto University Bioinformatics Center, Kyoto, Japan; https://www.genome.jp/kegg/) (accessed on 6 December 2024), and Pfam (European Bioinformatics Institute, Hinxton, Cambridgeshire, UK; http://pfam-legacy.xfam.org/) (accessed on 6 December 2024). Subsequently, defined collections of putative transcriptional regulators were manually curated for the selected strains’ genomes (Tables S1 and S2) and plotted using the CGView Server (version not specified), (Genome Context Tools, Bogotá, Colombia; http://genocat.tools/tools/cgview_server.html) (accessed on 6 December 2024). Moreover, to identify the common set of DNA-binding transcriptional regulators, comparative genomic analyses were performed.

### 3.2. Distribution of DNA-Binding Proteins

The search for *Pseudomonas* DNA-binding proteins was performed by means of the genome assignment server superfAMILY (https://supfam.org/SUPERFAMILY/) (University of Cambridge, United Kingdom), (accessed on 6 December 2024) that contains a library of hidden Markov models (HMMs) of the Pfam database based on the sequences of protein domains. To identify among the DNA-binding proteins those potentially representing transcriptional regulators, different HMM profiles of bacterial protein families with a known function in the transcriptional regulation of gene expression were downloaded from the Pfam database and used for searches against the predicted *Pseudomonas* proteins using hidden Markov model profiles. The HTH recognition tool designed by Dodd and Egan was used to scan the putative DNA-binding transcriptional regulators for the presence and position of HTH motifs by using PROSITE-Expasy, KEGG, and Pfam databases. Finally, the putative DNA-binding transcriptional regulators were grouped into regulatory protein families using the PANTHER knowledgebase (https://www.pantherdb.org/) (Stanford University, California, United States), (accessed on 6 December 2024).

### 3.3. Phylogenetic Analysis

Protein sequences with the same HTH domain were used to carry out a phylogenetic tree representation. All sequences were aligned by MUSCLE v5.2 software (https://www.ebi.ac.uk/jdispatcher/msa/muscle5?stype=protein) (accessed on 6 December 2024) and aligned proteins were used as inputs for the FastTree program (version 2.1.11) to build a phylogenetic tree using the JTT+CAT model. The resulting tree was plotted using iTool (iTOL v6) and annotated manually according to the individual protein annotation.

### 3.4. Classification Analysis

The functional classification of genes from the PAO1 and KT2440 strains was conducted by employing the Panther-GO plotting tool from PANTHER Classification System, version 17.0, based on PANTHER GO-slims and Gene Ontology terms (https://www.geneontology.org/), (accessed on 6 December 2024).

## 4. Conclusions

The bacterial TCSs play a key role in signaling, enabling the survival and colonization of pathogenic bacteria within their host. Given the urgent need for novel antimicrobial drugs, the regulatory function of TCSs makes them highly promising targets for the development of innovative therapeutics against bacterial infections. Due to their widespread presence and functional versatility, evaluating multiple compounds that target TCSs is essential. Numerous studies have documented both natural and synthetic compounds that exhibit a strong affinity for TCSs, demonstrating effective antimicrobial action against pathogenic bacteria. Therefore, a deeper understanding of the interactions between OCS and TCS and their targeted compound is critical. Such knowledge can contribute to refining the molecular structures of these compounds, thereby enhancing their specificity and effectiveness as ligands. Consequently, further research is needed to comprehensively elucidate the precise mechanisms of action of these drugs.

In this paper, we present a comprehensive analysis of transcriptional regulatory systems in both the non-pathogenic *P. putida* KT2440 and the pathogenic *P. aeruginosa* PAO1 strains. Additionally, we categorize and highlight the significance of TCSs in pathways related to virulence, resistance, and metabolism. Given that TCS-encoding genes are present in all Gram-positive and Gram-negative bacterial genomes, the development of a pharmacological TCS inhibitor that acts broadly and achieves the desired therapeutic effect would be exceptionally valuable in the fight against antibiotic resistance.

## Figures and Tables

**Table 1:** Categorization list of two-component system genes found in *P. putida* KT2440 and *P. aeruginosa* PAO1 and their corresponding families. nd: Not identified.

TCS Family	*P. putida* KT2440	*P. aeruginosa* PAO1	Key Functions
**OmpR family**			
PhoR-PhoB	PP_5321 (*phoR*)/PP_5320 (*phoB*)	PA5361 (*phoR*)/PA5360 (*phoB*)	Phosphate starvation response
PhoQ-PhoP	PP_1187 (*phoQ*)/PP_1186 (*phoP*)	PA1180 (*phoQ*)/PA1179 (*phoP*)	Magnesium transport
EnvZ-OmpR	PP_0247 (*envZ*)/PP_0246 (*ompR*)	PA5199 (*envZ*)/PA5200 (*ompR*)	Osmotic stress response
RstB-RstA	PP_1182 (*rstB*)/PP_1181 (*rstA*)	PA1158 (*rstB*)/PA1157 (*rstA*)	Envelope stress response
CpxA-CpxR	nd/PP_3372 (*cpxR*)	nd/PA3204 (*cpxR*)	Envelope stress response
CusS-CusR	PP_1437 (*cusS*)/PP_1438 (*czcR-III*), PP_2157 (*cusS*)/PP_2158 (*copR-I*), PP_5384 (*copS*)/PP_5383, (*copR-II*) Others: PP_0030 (*czrSA*)/nd	PA1438 (cusS)/PA1437 (*cusR*), PA4886 (*cusS*)/PA4885 (*cusR*), PA2524 (*cusS*)/PA2523 (*cusR*), PA2810 (*cusS*)/PA2809 (*cusR*)	Copper resistance and heavy metal tolerance
QseC-QseB	PP_2714 (*qseC*)/PP_2713 (*qseB*)	PA4777 (*qseC*)/PA4776 (*qseB*), PA2480 (*qseC*)/PA2479 (*qseB*)	Quorum-sensing
KdpD-KdpE	PP_4158 (*kdpD*)/PP_4157 (*kdpE*)	PA1636 (*kdpD*)/PA1637 (*kdpE*)	Potassium transport
TctE-TctD	PP_1421 (*tctE*)/PP_1420 (*tctD*)	PA0757 (*tctE*)/PA0756 (*tctD*)	Tricarboxylic acid transport
PfeS-PfeR	PP_0533 (*pfeS-I*)/PP_0534(*pfeR*), PP_1652 (*pfeSII)*/PP_1651 (*pfeR*)	PA2687 (*pfeS*)/PA2686 (*pfeR*), PA0930 (*pirS*)/PA0929 (*pirR*)	Iron acquisition
Unclassified	PP_3453 (nd)/PP_3454 (nd), PP_2403 (nd), PP_2907 (nd), PP_4224 (nd)/nd	PA3206 (*cpxS*)/nd, nd/PA4101 (*bfmR*), nd/PA2657 (*bqsR*), PA3191 (*gltS*)/PA3192 (*gltR*)	Miscellaneous roles in signal transduction and response regulation
**NarL family**			
UhpB-UhpA	PP_2671 (*uhpB*)/PP_0410 (*uhpA*)	PA1980 (*eraR*)/PA0410 (*uhpA*)	Hexose phosphate uptake
BarA-UvrY	PP_1650 (*gacS*)/nd, nd/PP_4099 (*uvrY*)	PA0928 (*gacS*)/PA2586 (*gacA*)	Central carbon metabolism regulation
EvgS-EvgA	PP_2100 (*evgS*)/PP_2101 (*evgA*), PP_3413 (*evgS*)/nd, nd/PP_1090 (*bvgA*)	PA3946 (*rocS1*)/PA3948(*rocA1*), PA2583 (*evgS*)/nd, nd/PA3045 (*evgA*)	Acid tolerance, drug resistance, virulence regulation
**LytTR family**			
AlgZ-AlgR	nd/PP_0185 (*algR*)	PA5262 (*algZ*)/PA5261 (*algR*)	Alginate biosynthesis regulation
**NtrC family**			
GlnL-GlnG	PP_5047 (*glnL*)/PP_5048 (*glnG*)	PA5124 (*ntrB*)/PA5125 (*ntrC*)	Nitrogen regulation
DctB-DctD	PP_0264 (nd)/PP_0263 (*dctD-I*), PP_1402 (dctB)/PP_1401 (*dctD-III*)	PA5165 (*dctB*)/PA5166 (*dctD*), PA5512 (*mifS*)/PA5511 (*mifR*)	C4-dicarboxylate transport regulation
KinB-AlgB	PP_0132 (*kinB*)/PP_0133 (*algB*)	PA5484 (*kinB*)/PA5483 (*algB*)	Alginate biosynthesis regulation
Unclassified	PP_2945 (*flgS*)/nd	PA2571 (*flgS*)/nd	Miscellaneous roles in signal transduction and response regulation
**CheA family**			
CheA-CheYBV	PP_4338 (*cheA*)/PP_4337 (*cheBA*), nd/PP_4340 (*cheY*), nd/PP_3759 (*cheB*), nd/PP_0802 (*cheV*), nd/PP_2128 (*cheV*), nd/PP_4393 (nd)	PA0178 (*cheA*)/nd, PA1458 (*cheA*)/PA1459 (*cheB*), nd/PA0179 (nd), nd/PA1456 (*cheY*), nd/PA0173 (nd), nd/PA3349 (*cheV*)	Chemotaxis
ChpA-ChpB/PilGH	nd/PP_4988 (nd), PP_4992 (*pilG*)/PP_4991 (*pilH*)	PA0413(*chpA*)/PA0414 (*chpB*), PA0408 (*pilG*)/PA0409 (*pilH*)	Chemosensory signal transduction and motility regulation
WspE-WspRF	PP_1492 (*wspE*)/PP_1494 (*wspR*)	PA3703 (*wspF*)/PA3702 (*wspR*), PA3704 (*wspE*)/PA3702 (*wspR*)	Chemosensory regulation; biofilm formation
Cph1-Rcp1	PP_2356 (*cph1*)/nd	nd/nd	Light-response regulation
**Other families**			
FlrB-FlrC	PP_4371 (*atoC*)/PP_4372 (*fleS*)	PA1098 (*fleS*)/PA1099 (*fleR*)	Flagellar synthesis regulation
AauS-AauR	PP_1066 (*dctD-II*)/PP_1067 (nd)	PA1335 (*auuR*)/PA1336 (*auuS*)	Acidic amino acid utilization regulation
RegB-RegA	PP_0887 (nd)/PP_0888 (*regA*)	PA4494 (*roxS*)/PA4493 (*roxR*)	Redox and oxidative stress response regulation
SagS-HptB-HsbR	PP_4362 (nd)/PP_4363 (nd), nd/PP_2664 (nd), nd/PP_1875 (nd), nd/PP_4173 (nd)	PA2824 (*sagS*)/nd, nd/PA1611 (nd), PA1976 (*ercS*)/nd, PA3345 (*hptB*)/PA3346 (*hsbR*)	Swarming, biofilm formation, and signaling
NasS-NasT	PP_2093 (*nasS*)/PP_2094 (*nasT*)	PA1786 (*nasS*)/PA1785 (*nasT*), nd/PA3363 (*amiR*)	Nitrate response regulation
Unclassified	PP_4781 (nd)/PP_4824 (nd)	PA3974 (*ladS*)/PA4856 (*retS*)	

**Table 2:** Categorization of virulence genes in *P. aeruginosa* PAO1 and *P. putida* KT2440. nd: Not identified.

Virulence Factors	Gene Name	*P. aeruginosa* PAO1	*P. putida* KT2440
**Adherence**			
**Type IV pili**	*pilA*	PA4525	PP_0634
	*pilB*	PA4526	nd
	*pilC*	PA4527	PP_0633
	*pilD*	PA4528	PP_0632
	*pilE*	PA4556	PP_0611
	*pilF*	PA3805	PP_0851
	*pilM*	PA5044	PP_5083
	*pilN*	PA5043	PP_5082
	*pilO*	PA5042	nd
	*pilP*	PA5041	PP_5081
	*pilQ*	PA5040	PP_5080
	*pilT*	PA0395	PP_5093
	*pilU*	PA0396	nd
	*pilV*	PA4551	nd
	*pilW*	PA4552	nd
	*pilX*	PA4553	nd
	*pilY1*	PA4554	nd
	*pilY2*	PA4555	nd
	*pilZ*	PA2960	nd
	*fimT*	PA4549	nd
	*fimU*	PA4550	nd
	*fimV*	PA3115	PP_1993
	*pilR*	PA4547	nd
	*pilS*	PA4546	nd
	*pilG*	PA0408	PP_4992
	*pilH*	PA0409	PP_4991
	*pilI*	PA0410	PP_4990
	*pilJ*	PA0411	PP_4989
	*pilK*	PA0412	nd
	*chpA*	PA0413	PP_4988
	*chpB*	PA0414	nd
	*chpC*	PA0415	PP_4987
	*chpD*	PA0416	nd
	*chpE*	PA0417	nd
**Effector delivery system**			
**Exolysin**	*exlA*	nd	PP_1449
	*exlB*	nd	PP_1450
**HSI-1**	*tagR*	PA0071	nd
	*tagS*	PA0072	nd
	*tagT*	PA0073	nd
	*ppkA*	PA0074	nd
	*pppA*	PA0075	nd
	*tagF*	PA0076	nd
	*icmF1*	PA0077	nd
	*dotU1*	PA0078	nd
	*hsiJ1*	PA0079	nd
	*lip1*	PA0080	nd
	*fha1*	PA0081	nd
	*hsiA1*	PA0082	nd
	*hsiB1*	PA0083	nd
	*hsiC1*	PA0084	nd
	*hcp1*	PA0085	nd
	*hsiE1*	PA0086	nd
	*hsiF1*	PA0087	nd
	*hsiG1*	PA0088	nd
	*hsiH1*	PA0089	nd
	*clpV1*	PA0090	nd
	*vgrG1a*	PA0091	nd
**HSI-1 T6SS**	*tse1*	PA1844	nd
	*tse2*	PA2702	nd
	*tse3*	PA3484	nd
	*tse7*	PA0099	nd
	*tse4*	PA2774	nd
	*tse5*	PA2684	nd
	*tse6*	PA0093	nd
**HSI-2**	*vgrG*	PA1511	nd
	*hcpA*	PA1512	nd
	*tssA*	PA1656	nd
	*tssB*	PA1657	nd
	*tssC*	PA1658	nd
	*tssE*	PA1659	nd
	*tssF*	PA1660	nd
	*tssG*	PA1661	nd
	*tssH*	PA1662	nd
	*tssJ*	PA1666	nd
	*tssK*	PA1667	nd
	*icmH*	PA1668	nd
	*tssM*	PA1669	nd
**HSI-2 T6SS**	*pldA*	PA3487	nd
	*vgrG2b*	PA0262	nd
**HSI-3**	*tssA*	PA2360	nd
	*tssM*	PA2361	nd
	*icmH*	PA2362	nd
	*tssK*	PA2363	nd
	*tssB*	PA2365	nd
	*tssC*	PA2366	nd
	*hcp*	PA2367	nd
	*tssE*	PA2368	nd
	*tssF*	PA2369	nd
	*tssG*	PA2370	nd
	*tssH*	PA2371	nd
	*tssI*	PA2373	nd
**HSI-3 T6SS**	*pldB*	PA5089	nd
**LasA**	*lasA*	PA1871	nd
**LasB**	*lasB*	PA3724	nd
**Putida K1-T6SS**	*tssA1*	nd	PP_3088
	*hcp1*	nd	PP_3089
	*tssM1*	nd	PP_3090
	*tagF1*	nd	PP_5561
	*tssL1*	nd	PP_3092
	*tssK1*	nd	PP_3093
	*tssJ1*	nd	PP_3094
	*tssH*	nd	PP_3095
	*tssG1*	nd	PP_3096
	*tssF1*	nd	PP_3097
	*tssE1*	nd	PP_3098
	*tssC1*	nd	PP_3099
	*tssB1*	nd	PP_3100
	*vgrG1*	nd	PP_3106
**Putida K2-T6SS**	*tssM2*	nd	PP_4071
	*tssA2*	nd	PP_4072
	*vasl2*	nd	PP_4073
	*tssB2*	nd	PP_4074
	*tssE2*	nd	PP_4076
	*tssF2*	nd	PP_4077
	*tssG2*	nd	PP_4078
	*tssJ2*	nd	PP_4079
	*tssK2*	nd	PP_4080
	*tssL2*	nd	PP_4081
	*hcp2*	nd	PP_4082
**Putida K3-T6SS**	*vgrG3*	nd	PP_2614
	*hcp3*	nd	PP_2615
	*tssL3*	nd	PP_2616
	*tssK3*	nd	PP_2617
	*tssJ3*	nd	PP_2618
	*fha3*	nd	PP_2619
	*tssG3*	nd	PP_2620
	*tssF3*	nd	PP_2621
	*tssE3*	nd	PP_2622
	*tssC3*	nd	PP_2623
	*tssB3*	nd	PP_2624
	*vasl3*	nd	PP_2625
	*tssA3*	nd	PP_2626
	*tssM3*	nd	PP_2627
**Putida-T6SS**	*tke1*	nd	PP_3103
	*tke2*	nd	PP_3108
	*tke4*	nd	PP_4085
	*tke5*	nd	PP_2612
	*tke6*	nd	PP_0646
	*tke7*	nd	PP_4885
	*tke9*	nd	PP_3388
	*tke10*	nd	PP_4048
**TTSS**	*pscU*	PA1690	nd
	*pscT*	PA1691	nd
	*pscS*	PA1692	nd
	*pscR*	PA1693	nd
	*pscQ*	PA1694	nd
	*pscP*	PA1695	nd
	*pscO*	PA1696	nd
	*pscN*	PA1697	nd
	*popN*	PA1698	nd
	*pcr1*	PA1699	nd
	*pcr2*	PA1700	nd
	*pcr3*	PA1701	nd
	*pcr4*	PA1702	nd
	*pcrD*	PA1703	nd
	*pcrR*	PA1704	nd
	*pcrG*	PA1705	nd
	*pcrV*	PA1706	nd
	*pcrH*	PA1707	nd
	*popB*	PA1708	nd
	*popD*	PA1709	nd
	*exsC*	PA1710	nd
	*exsE*	PA1711	nd
	*exsB*	PA1712	nd
	*exsA*	PA1713	nd
	*exsD*	PA1714	nd
	*pscB*	PA1715	nd
	*pscC*	PA1716	nd
	*pscD*	PA1717	nd
	*pscE*	PA1718	nd
	*pscF*	PA1719	nd
	*pscG*	PA1720	nd
	*pscH*	PA1721	nd
	*pscI*	PA1722	nd
	*pscJ*	PA1723	nd
	*pscK*	PA1724	nd
	*pscL*	PA1725	nd
**TTSS effector proteins**	*exoS*	PA3841	nd
	*exoT*	PA0044	nd
	*exoY*	PA2191	nd
**Motility**			
**Flagella**	*flgB*	PA1077	PP_4391
	*flgC*	PA1078	PP_4390
	*flgD*	PA1079	PP_4389
	*flgE*	PA1080	PP_4388
	*flgF*	PA1081	PP_4386
	*flgG*	PA1082	PP_4385
	*flgH*	PA1083	PP_4384
	*flgI*	PA1084	PP_4383
	*flgJ*	PA1085	PP_4382
	*flgK*	PA1086	PP_4381
	*flgL*	PA1087	PP_4380
	*fliC*	PA1092	PP_4378
	*fleI*	PA1093	PP_4377
	*fliD*	PA1094	PP_4376
	*fliS*	PA1095	PP_4375
	*fleP*	PA1096	PP_4374
	*fleQ*	PA1097	PP_4373
	*fleS*	PA1098	PP_4372
	*fleR*	PA1099	PP_4371
	*fliE*	PA1100	PP_4370
	*fliF*	PA1101	PP_4369
	*fliG*	PA1102	PP_4368
	*fliH*	PA1103	PP_4367
	*fliI*	PA1104	PP_4366
	*fliJ*	PA1105	PP_4365
	*fliK*	PA1441	PP_4361
	*fliL*	PA1442	PP_4359
	*fliM*	PA1443	PP_4358
	*fliN*	PA1444	PP_4357
	*fliO*	PA1445	PP_4356
	*fliP*	PA1446	PP_4355
	*fliQ*	PA1447	PP_4354
	*fliR*	PA1448	PP_4353
	*flhB*	PA1449	PP_4352
	*flhA*	PA1452	PP_4344
	*flhF*	PA1453	PP_4343
	*fleN*	PA1454	PP_4342
	*fliA*	PA1455	PP_4341
	*flgA*	PA3350	PP_4394
	*flgM*	PA3351	PP_4395
	*flgN*	PA3352	PP_4396
	*motB*	PA4953	PP_4904
	*motA*	PA4954	PP_4905
	*motC*	PA1460	PP_4336
	*motD*	PA1461	PP_4335
	*motY*	PA3526	PP_1087
**Exotoxin**			
**ExoA**	*toxA*	PA1148	nd
**Non-hemolytic phospholipase C**	*plcN*	PA3319	nd
**Phospholipase C**	*plcB*	PA0026	nd
**PLC**	*plcH*	PA0844	nd
**Exoenzyme**			
**Alkaline protease**	*aprA*	PA1249	nd
**Protease IV**	*prpL*	PA4175	nd
**Immune modulation**			
**Lipopolysaccharide (LPS)**	*nd*	PA3141	nd
		PA3142	nd
		PA3143	nd
		PA3145	nd
		PA3146	nd
		PA3147	nd
		PA3148	nd
		PA3149	nd
		PA3150	nd
		PA3151	nd
		PA3152	nd
		PA3153	nd
		PA3154	nd
		PA3155	nd
		PA3156	nd
		PA3157	nd
		PA3158	nd
		PA3160	nd
**Rhamnolipid**	*rhlA*	PA3479	nd
	*rhlB*	PA3478	nd
	*rhlC*	PA1130	nd
**Biofilm**			
**Acylhomoserine lactone synthase**	*hdtS*	PA0005	PP_0058
**Alginate**	*algD*	PA3540	PP_1288
	*alg8*	PA3541	PP_1287
	*alg44*	PA3542	PP_1286
	*algK*	PA3543	PP_1285
	*algE*	PA3544	PP_1284
	*algG*	PA3545	PP_1283
	*algX*	PA3546	PP_1282
	*algL*	PA3547	PP_1281
	*algI*	PA3548	PP_1280
	*algJ*	PA3549	PP_1279
	*algF*	PA3550	PP_1278
	*algA*	PA3551	PP_1277
	*algC*	PA5322	PP_5288
	*algU*	PA0762	PP_1427
	*mucA*	PA0763	PP_1428
	*mucB*	PA0764	PP_1429
	*mucC*	PA0765	nd
	*mucD*	PA0766	PP_1430
	*algR*	PA5261	PP_0185
	*algZ*	PA5262	nd
	*algW*	PA4446	PP_1301
	*mucE*	PA4033	nd
	*mucP*	PA3649	PP_1598
	*algP/algR3*	PA5253	PP_0194
	*algQ*	PA5255	PP_0191
**Quorum-sensing**			
	*rhlR*	PA3477	nd
	*rhlI*	PA3476	nd
	*lasR*	PA1430	nd
	*lasI*	PA1432	nd
**Nutritional/Metabolic factor**			
**Pyochelin**	*pchI*	PA4222	nd
	*pchH*	PA4223	nd
	*pchG*	PA4224	nd
	*pchF*	PA4225	nd
	*pchE*	PA4226	nd
	*pchR*	PA4227	nd
	*pchD*	PA4228	nd
	*pchC*	PA4229	nd
	*pchB*	PA4230	nd
	*pchA*	PA4231	nd
	*fptA*	PA4221	nd
**Pyocyanin**	*phzA1*	PA4210	nd
	*phzB1*	PA4211	nd
	*phzC1*	PA4212	nd
	*phzD1*	PA4213	nd
	*phzE1*	PA4214	nd
	*phzF1*	PA4215	nd
	*phzG1*	PA4216	nd
	*phzA2*	PA1899	nd
	*phzB2*	PA1900	nd
	*phzC2*	PA1901	nd
	*phzD2*	PA1902	nd
	*phzE2*	PA1903	nd
	*phzF2*	PA1904	nd
	*phzG2*	PA1905	nd
	*phzM*	PA4209	nd
	*phzS*	PA4217	nd
	*phzH*	PA0051	nd
**Pyoverdine**	*pvdQ*	PA2385	PP_2901
	*pvdA*	PA2386	PP_3796
	*pvdP*	PA2392	PP_4212
	*pvdM*	PA2393	PP_4213
	*pvdN*	PA2394	PP_4214
	*pvdO*	PA2395	PP_4215
	*pvdF*	PA2396	nd
	*pvdE*	PA2397	PP_4216
	*pvdD*	PA2399	PP_4219
	*pvdJ*	PA2400	nd
	*pvdI*	PA2402	nd
	*pvdH*	PA2413	PP_4223
	*pvdL*	PA2424	PP_4243
	*pvdG*	PA2425	nd
	*pvdS*	PA2426	PP_4244
	*pvdY*	PA2427	PP_4245
	*fpvA*	PA2398	PP_4217
**Antimicrobial activity/Competitive advantage**			
**Hydrogen cyanide production**	*hcnA*	PA2193	nd
	*hcnB*	PA2194	nd
	*hcnC*	PA2195	nd
**Regulation**			
**GacS/GacA**	*gacS*	PA0928	PP_1650
	*gacA*	PA2586	PP_4099

## Data Availability

The original contributions presented in this study are included in the article. Further inquiries can be directed to the corresponding author.

## Supplementary Materials

The following supporting information can be downloaded at: https://www.mdpi.com/article/10.3390/ijms26104677/s1.
