# Supplementary material for: Transcriptional Regulatory Systems in Pseudomonas: A Comparative Analysis of Helix-Turn-Helix Domains and Two-Component Signal Transduction Networks
Source: Int J Mol Sci. 2025 May 14;26(10):4677. doi: 10.3390/ijms26104677 (PMC12112638; doi:10.3390/ijms26104677)
Supplement: Supplementary file 1 [file ijms-26-04677-s001.zip › Table S3_IJMS_NV.pdf]

Suppl S3: Similarity categorization of transcriptional regulators in *P. putida* KT2440 and *P. aeruginosa* PAO1 into Regulatory Protein Families.

***Pseudomonas putida* KT2440**	***Pseudomonas aeruginosa* PAO1**
**AraC family**	
PP_3538	PA4120
PP_2173	PA0416
PP_4605	PA4227
PP_0706	PA0248
PP_0305	PA0564
PP_4482	PA2332
PP_0298	PA3898
PP_3665	PA2696
	PA3596
	PA2118
	PA5389
	PA0893
	PA5380
	PA4184
	PA1713
**LysR family**	
PP_3143	PA3845
PP_0661	PA1184
PP_1393	PA0784
PP_4107	PA2383
PP_1713	PA4914
PP_1063	PA5293
PP_2444	PA0152
PP_5309	PA3587
PP_0917	PA5344
PP_2327	PA4363
PP_3121	PA2054
PP_2516	PA1754
PP_2826	PA0217
PP_2074	PA2921
PP_3669	PA1998
PP_5337	PA4109
PP_0595	PA2492
PP_5348	PA2056
PP_3716	PA1003
PP_2350	PA5428
	PA0133
	PA1145
	PA1422
	PA5437
	PA2510
	PA1853
**DeoR family**	
PP_1074	PA3583
	PA1490
**LacI family**	
PP_2457	PA1949
PP_3380	PA2259
PP_0792	PA3563
PP_3415	PA2320
**GntR family**	
PP_5035	PA3381
PP_0204	PA5105
PP_1697	PA3757
PP_4734	PA2299
PP_3603	PA4769
PP_2254	PA0120
PP_3744	PA5356

PP_3750	PA0268
PP_5342	PA5431
PP_2642	PA4165
PP_3738	PA4906
PP_2908	
PP_3649	
**TetR/AcrR family**	
PP_5719	PA5374
PP_1387	PA0167
PP_0594	PA0436
PP_4295	PA1504
PP_4039	PA1864
PP_2475	PA2196
PP_1515	PA0839
PP_2820	PA1241
PP_3949	PA2766
	PA4831
	PA3678
	PA2020
	PA3721
	PA3574
	PA4600
	PA0475
	PA4890
	PA2885
**Lrp/AsnC family**	
PP_5271	PA2577
PP_4308	PA5308
PP_4776	PA3965
PP_4424	PA4508
PP_4400	PA4784
PP_1307	PA2246
PP_4595	PA2028
PP_5188	
**LuxR family**	
PP_0767	PA1759
	PA3921
	PA1760
	PA1430
	PA3477
	PA1136
**MarR family**	
PP_4515	PA0253
PP_0175	PA3341
PP_3946	PA0424
PP_1860	PA1603
	PA2849
	PA2825
**MerR family**	
PP_0585	PA4778
PP_2060	PA2273
PP_2740	PA4659
PP_4630	
PP_0740	
**Fur family**	
PP_4730	PA4764
PP_0119	PA2384
	PA5499
**IclR family**	
PP_1375	PA0236
PP_2609	PA0155
**CRP/FNR family**	
PP_0424	PA0652
PP_4265	PA0275

PP_3287	PA1544
	PA0527
**Rrf2 family**	
PP_0841	PA3815
**ArsR family**	
PP_1930	PA2277
PP_2718	PA2277
PP_2718	
**Other families**	
PP_5350	PA5438
PP_1021	PA3184
PP_0437	PA4280
PP_4821	PA4853
PP_2790	PA1097
PP_4204	PA4147
PP_0274	PA4021
PP_4373	PA2359
PP_2259	PA1663
PP_3467	PA2665
PP_0557	PA1050
PP_0546	PA1196
PP_0807	PA0487
PP_3177	PA3864
PP_0360	PA2785
PP_3192	PA5403
PP_1198	PA4077
PP_4947	PA0782
**CopG family**	
PP_3341	
**Other transcription factors**	
PP_1757	PA3391
PP_0001	PA0857
PP_3186	PA5562
PP_4489	PA0873
PP_1236	PA2449
PP_3286	PA1009
PP_5223	PA5274
PP_4997	PA0403
PP_3238	PA4182
PP_5527	PA0405
PP_5343	PA4723
PP_4995	PA5536
PP_4693	PA4057
PP_2220	PA4581
PP_0513	PA4421
PP_1328	
