# Supplementary material for: Transcriptional Regulatory Systems in Pseudomonas: A Comparative Analysis of Helix-Turn-Helix Domains and Two-Component Signal Transduction Networks
Source: Int J Mol Sci. 2025 May 14;26(10):4677. doi: 10.3390/ijms26104677 (PMC12112638; doi:10.3390/ijms26104677)
Supplement: Supplementary file 1 [file ijms-26-04677-s001.zip › Table S4_IJMS_NV.pdf]

Suppl. S4: List of *P. putida* KT2440 transcriptional regulators found in *P. aeruginosa* PAO1 and the corresponding KEGG Orthology (KO term).

Gene	KO term	Annotation
PP_3538	K18954	pobR; AraC family, transcriptional activator of pobA
PP_4605	K07506	AraC family transcriptional regulator
PP_0706	K10778	ada; AraC family transcriptional regulator, regulatory protein of adaptative response / methylated-DNA-[protein]-cysteine methyltransferase
PP_0305	K17736	cdhR; AraC family transcriptional regulator, carnitine catabolism transcriptional activator
PP_4482	K21825	argR; AraC family transcriptional regulator, L-arginine-responsive activator
PP_0298	K21826	gbdR; AraC family transcriptional regulator, glycine betaine-responsive activator
PP_3665	K21826	gbdR; AraC family transcriptional regulator, glycine betaine-responsive activator
PP_3143	K03566	gcvA; LysR family transcriptional regulator, glycine cleavage system transcriptional activator
PP_0661	K03566	LysR family transcriptional regulator, glycine cleavage system transcriptional activator
PP_1393	K03566	LysR family transcriptional regulator, glycine cleavage system transcriptional activator
PP_4107	K03566	LysR family transcriptional regulator, glycine cleavage system transcriptional activator
PP_1713	K02623	pcaQ; LysR family transcriptional regulator, pca operon transcriptional activator
PP_1063	K03576	metR; LysR family transcriptional regulator, regulator for metE and metH
PP_5309	K04761	oxyR; LysR family transcriptional regulator, hydrogen peroxide-inducible genes activator
PP_0917	K05596	iciA; LysR family transcriptional regulator, chromosome initiation inhibitor
PP_2327	K13634	cysB; LysR family transcriptional regulator, cys regulon transcriptional activator
PP_3121	K17737	dhcR; LysR family transcriptional regulator, carnitine catabolism transcriptional activator
PP_2826	K18297	mexT; LysR family transcriptional regulator, mexEF-oprN operon transcriptional activator
PP_2074	K18297	LysR family transcriptional regulator, mexEF-oprN operon transcriptional activator
PP_3669	K18297	LysR family transcriptional regulator, mexEF-oprN operon transcriptional activator
PP_5337	K21645	hypT; LysR family transcriptional regulator, hypochlorite-specific transcription factor HypT
PP_0595	K21699	bauR; LysR family transcriptional regulator, transcriptional activator for bauABCD operon
PP_5348	K21711	pycR; LysR family transcriptional regulator, putative pyruvate carboxylase regulator
PP_3716	K21757	catR; LysR family transcriptional regulator, benzoate and cis,cis-muconate-responsive activator of ben and cat genes
PP_2350	K23773	hdfR; LysR family transcriptional regulator, flagellar master operon regulator
PP_1074	K02444	glpR; DeoR family transcriptional regulator, glycerol-3-phosphate regulon repressor
PP_2457	K02529	rbsR; lacI; LacI family transcriptional regulator
PP_3380	K02525	ptxS, LacI family transcriptional regulator, kdg operon repressor

PP_0792	K03435	cra; LacI family transcriptional regulator, fructose operon transcriptional repressor
PP_3415	K06145	gntR; LacI family transcriptional regulator, gluconate utilization system Gnt-I transcriptional repressor
PP_5035	K05836	hutC; GntR family transcriptional regulator, histidine utilization repressor
PP_0204	K03710	GntR family transcriptional regulator
PP_1697	K03710	K03710; GntR family transcriptional regulator
PP_4734	K05799	pdhR; GntR family transcriptional regulator, transcriptional repressor for pyruvate dehydrogenase complex
PP_3603	K05799	GntR family transcriptional regulator,
PP_2254	K05799	GntR family transcriptional regulator,
PP_3744	K11474	glcC; GntR family transcriptional regulator, glc operon transcriptional activator
PP_3750	K00375	GntR family transcriptional regulator / MocR family aminotransferase
PP_5342	K00375	GntR family transcriptional regulator / MocR family aminotransferase
PP_2642	K00375	GntR family transcriptional regulator / MocR family aminotransferase
PP_3738	K11475	vanR; GntR family transcriptional regulator, vanillate catabolism transcriptional regulator
PP_5719	K02167	betI; TetR/AcrR family transcriptional regulator, transcriptional repressor of bet genes
PP_0594	K09017	TetR/AcrR family transcriptional regulator
PP_4295	K09017	TetR/AcrR family transcriptional regulator
PP_4039	K09017	rutR; TetR/AcrR family transcriptional regulator
PP_2475	K16137	nemR; TetR/AcrR family transcriptional regulator, transcriptional repressor for nem operon
PP_1515	K18301	mexL; TetR/AcrR family transcriptional regulator, mexJK operon transcriptional repressor
PP_2820	K18294	nfxB; TetR/AcrR family transcriptional regulator, mexCD-oprJ operon repressor
PP_5271	K03719	lrp; Lrp/AsnC family transcriptional regulator, leucine-responsive regulatory protein
PP_4308	K03719	lrp; Lrp/AsnC family transcriptional regulator, leucine-responsive regulatory protein
PP_4776	K03719	AsnC; Lrp/AsnC family transcriptional regulator, leucine-responsive regulatory protein
PP_4424	K03719	Uncharacterized HTH-type transcriptional regulator
PP_4400	K03719	bkdR; Lrp/AsnC family transcriptional regulator, leucine-responsive regulatory protein
PP_1307	K03719	AsnC family transcriptional regulator, leucine-responsive regulatory protein
PP_4595	K05800	ybaO; Lrp/AsnC family transcriptional regulator
PP_5188	K05800	Lrp/AsnC family transcriptional regulator
PP_0767	K03556	malT; LuxR family transcriptional regulator, maltose regulon positive regulatory protein
PP_4515	K06075	slyA; MarR family transcriptional regulator, transcriptional regulator for hemolysin
PP_0175	K06075	MarR family transcriptional regulator, transcriptional regulator for hemolysin
PP_3946	K22296	nicR; MarR family transcriptional regulator, lower aerobic nicotinate degradation pathway regulator
PP_1860	K23775	ohrR; MarR family transcriptional regulator, organic hydroperoxide resistance regulator
PP_0585	K19591	cueR; MerR family transcriptional regulator, copper efflux regulator

PP_2060	K13639	soxR; MerR family transcriptional regulator, redox-sensitive transcriptional activator SoxR
PP_2740	K22491	litR; MerR family transcriptional regulator, light-induced transcriptional regulator
PP_4630	K22491	MerR family transcriptional regulator, light-induced transcriptional regulator
PP_0740	K22491	MerR family transcriptional regulator, light-induced transcriptional regulator
PP_4730	K03711	fur; Fur family transcriptional regulator, ferric uptake regulator
PP_0119	K09823	zur; Fur family transcriptional regulator, zinc uptake regulator
PP_1375	K02624	pcaR; IclR family transcriptional regulator, pca regulon regulatory protein
PP_2609	K02624	IclR family transcriptional regulator, pca regulon regulatory protein
PP_0424	K10914	crp; CRP/FNR family transcriptional regulator, cyclic AMP receptor protein
PP_4265	K01420	fnr; CRP/FNR family transcriptional regulator, anaerobic regulatory protein
PP_3287	K01420	fnrC; CRP/FNR family transcriptional regulator, anaerobic regulatory protein
PP_0841	K13643	iscR; Rrf2 family transcriptional regulator, iron-sulfur cluster assembly transcription factor
PP_1930	K03892	arsR; ArsR family transcriptional regulator, arsenate/arsenite/antimonite-responsive transcriptional repressor
PP_2718	K03892	arsR-II; ArsR family transcriptional regulator, arsenate/arsenite/antimonite-responsive transcriptional repressor
PP_5350	K19337	RpiR family transcriptional regulator, carbohydrate utilization regulator
PP_1021	K19337	hexR; RpiR family transcriptional regulator, carbohydrate utilization regulator
PP_0437	K03524	birA; BirA family transcriptional regulator, biotin operon repressor / biotin---[acetyl-CoA-carboxylase] ligase
PP_4821	K03557	Fis family transcriptional regulator, factor for inversion stimulation protein
PP_4373	K10941	fleQ; sigma-54 dependent transcriptional regulator, flagellar regulatory protein
PP_2259	K21405	sigma-54 dependent transcriptional regulator, acetoin dehydrogenase operon transcriptional activator AcoR
PP_3467	K21405	sigma-54 dependent transcriptional regulator, acetoin dehydrogenase operon transcriptional activator AcoR
PP_0557	K21405	acoR; sigma-54 dependent transcriptional regulator, acetoin dehydrogenase operon transcriptional activator AcoR
PP_0546	K21405	sigma-54 dependent transcriptional regulator, acetoin dehydrogenase operon transcriptional activator AcoR
PP_0807	K12266	norR; anaerobic nitric oxide reductase transcription regulator
PP_3177	K02647	hypothetical protein; cdaR; carbohydrate diacid regulator
PP_0360	K02019	modR; molybdate transport system regulatory protein
PP_3192	K21829	dauR; D-arginine utilization repressor
PP_4947	K13821	putA; RHH-type transcriptional regulator, proline utilization regulon repressor / proline dehydrogenase / delta 1-pyrroline-5-carboxylate dehydrogenase
PP_1757	K05527	bolA; BolA family transcriptional regulator, general stress-responsive regulator
PP_0001	K03497	parB; ParB family transcriptional regulator, chromosome partitioning protein
PP_4489	K03721	phhR; tyrR; transcriptional regulator of aroF, aroG, tyrA and aromatic amino acid transport
PP_1236	K03567	gcvR; glycine cleavage system transcriptional repressor
PP_5223	K06140	rnk; regulator of nucleoside diphosphate kinase

PP_4997	K02825	pyrR; pyrimidine operon attenuation protein / uracil phosphoribosyltransferase
PP_3238	K02825	pyrimidine operon attenuation protein / uracil phosphoribosyltransferase
PP_5343	K07734	paiB; transcriptional regulator
PP_4995	K07735	algH; putative protein possibly involved in the control of exopolysaccharide production
PP_4693	K06204	dksA; DnaK suppressor protein
PP_2220	K06204	dksA; C4-type zinc finger protein, DnaK suppressor protein
PP_0513	K07738	nrdR; DNA-binding transcriptional repressor NrdR-Zn2+-ATP/dATP
PP_1328	K03925	mraZ; transcriptional regulator MraZ family

List of P. aeruginosa PAO1 transcriptional regulators found in P. putida KT2440 and the corresponding KEGG Orthology (KO term).

Gene	KO term	Annotation
PA0248	K18954	pobR; AraC family , transcriptional activator of pobA
PA3898	K07506	AraC family
PA2696	K07506	AraC family
PA3596	K10778	ada; AraC family , regulatory protein of adaptative response / methylated-DNA-[protein]-cysteine methyltransferase
PA2118	K10778	ada; AraC family , regulatory protein of adaptative response / methylated-DNA-[protein]-cysteine methyltransferase
PA5389	K17736	cdhR; AraC family , carnitine catabolism transcriptional activator
PA0893	K21825	argR; AraC family , L-arginine-responsive activator
PA5380	K21826	gbdR; AraC family , glycine betaine-responsive activator
PA4184	K21826	gbdR; AraC family , glycine betaine-responsive activator
PA3845	K03566	gcvA; LysR family , glycine cleavage system transcriptional activator
PA1184	K03566	gcvA; LysR family , glycine cleavage system transcriptional activator
PA0784	K03566	gcvA; LysR family , glycine cleavage system transcriptional activator
PA2383	K03566	gcvA; LysR family , glycine cleavage system transcriptional activator
PA4914	K03566	gcvA; LysR family , glycine cleavage system transcriptional activator
PA5293	K03566	gcvA; LysR family , glycine cleavage system transcriptional activator
PA0152	K02623	pcaQ; LysR family , pca operon transcriptional activator
PA3587	K03576	metR; LysR family , regulator for metE and metH
PA5344	K04761	oxyR; LysR family , hydrogen peroxide-inducible genes activator
PA4363	K05596	iciA; LysR family , chromosome initiation inhibitor
PA1754	K13634	cysB; LysR family , cys regulon transcriptional activator
PA1998	K17737	dhcR; LysR family , carnitine catabolism transcriptional activator
PA2492	K18297	mexT; LysR family , mexEF-oprN operon transcriptional activator
PA5428	K21645	hypT; LysR family , hypochlorite-specific transcription factor HypT
PA0133	K21699	bauR; LysR family , transcriptional activator for bauABCD operon
PA1145	K21699	bauR; LysR family , transcriptional activator for bauABCD operon
PA1422	K21699	bauR; LysR family , transcriptional activator for bauABCD operon
PA5437	K21711	pycR; LysR family , putative pyruvate carboxylase regulator
PA2510	K21757	benM; LysR family , benzoate and cis,cis-muconate-responsive activator
PA1853	K23773	hdfR; LysR family , flagellar master operon regulator
PA3583	K02444	glpR; DeoR family , glycerol-3-phosphate regulon repressor
PA1490	K02444	glpR; DeoR family , glycerol-3-phosphate regulon repressor
PA1949	K02529	rbsR; ribose operon repressor RbsR. lacI; LacI family
PA2259	K02525	PtxS; LacI family , kdg operon repressor
PA3563	K03435	fruR1; LacI family , fructose operon transcriptional repressor
PA2320	K06145	gntR; LacI family , transcriptional repressor gluconate utilization system
PA5105	K05836	hutC; GntR family , histidine utilization repressor
PA2299	K03710	K03710; GntR family

PA4769	K05799	pdhR; GntR family , transcriptional repressor for pyruvate dehydrogenase complex
PA0120	K05799	pdhR; GntR family , transcriptional repressor for pyruvate dehydrogenase complex
PA5356	K11474	glcC; GntR family , glc operon transcriptional activator
PA0268	K00375	K00375; GntR family / MocR family aminotransferase
PA5431	K00375	K00375; GntR family / MocR family aminotransferase
PA4165	K00375	K00375; GntR family / MocR family aminotransferase
PA4906	K11475	vanR; GntR family , vanillate catabolism
PA5374	K02167	betI; TetR/AcrR family , transcriptional repressor of bet genes
PA0167	K09017	rutR; TetR/AcrR family
PA0436	K09017	rutR; TetR/AcrR family
PA1504	K09017	rutR; TetR/AcrR family
PA1864	K09017	rutR; TetR/AcrR family
PA2196	K16137	nemR; TetR/AcrR family , transcriptional repressor for nem operon
PA0839	K16137	nemR; TetR/AcrR family , transcriptional repressor for nem operon
PA1241	K16137	nemR; TetR/AcrR family , transcriptional repressor for nem operon
PA2766	K16137	nemR; TetR/AcrR family , transcriptional repressor for nem operon
PA4831	K16137	nemR; TetR/AcrR family , transcriptional repressor for nem operon
PA3678	K18301	mexL; TetR/AcrR family , mexJK operon transcriptional repressor
PA4600	K18294	nfxB; TetR/AcrR family , mexCD-oprJ operon repressor
PA2577	K03719	lrp; Lrp/AsnC family , leucine-responsive regulatory protein
PA5308	K03719	lrp; Lrp/AsnC family , leucine-responsive regulatory protein
PA3965	K03719	lrp; Lrp/AsnC family , leucine-responsive regulatory protein
PA4508	K03719	lrp; Lrp/AsnC family , leucine-responsive regulatory protein
PA4784	K03719	lrp; Lrp/AsnC family , leucine-responsive regulatory protein
PA2246	K03719	Bkd; Lrp/AsnC family , leucine-responsive regulatory protein
PA2028	K05800	ybaO; Lrp/AsnC family
PA1759	K03556	malT; LuxR family , maltose regulon positive regulatory protein
PA3921	K03556	malT; LuxR family , maltose regulon positive regulatory protein
PA1760	K03556	malT; LuxR family , maltose regulon positive regulatory protein
PA3341	K06075	slyA; MarR family , for hemolysin
PA1603	K22296	nicR; MarR family , lower aerobic nicotinate degradation pathway regulator
PA2849	K23775	ohrR; MarR family , organic hydroperoxide resistance regulator
PA2825	K23775	ospR; MarR family
PA4778	K19591	cueR; MerR family , copper efflux regulator
PA2273	K13639	soxR; redox-sensitive transcriptional activator
PA4659	K22491	litR; MerR family , light-induced
PA4764	K03711	fur; ferric uptake regulation protein, Fur family
PA2384	K03711	hypothetical protein
PA5499	K09823	zur; Fur family , zinc uptake regulator
PA0155	K02624	pcaR; IclR family , pca regulon regulatory protein
PA0652	K10914	vfr; cAMP-regulatory protein; CRP/FNR family , cyclic AMP receptor protein
PA0275	K10914	crp; CRP/FNR family , cyclic AMP receptor protein
PA1544	K01420	Anr; CRP/FNR family , anaerobic regulatory protein
PA3815	K13643	iscR; Rrf2 family , iron-sulfur cluster assembly transcription factor
PA2277	K03892	arsR; ArsR family , arsenate/arsenite/antimonite-responsive transcriptional repressor
PA5438	K19337	RpiR family , carbohydrate utilization regulator
PA3184	K19337	hexR; RpiR family , carbohydrate utilization regulator
PA4280	K03524	birA; BirA family , biotin operon repressor
PA4853	K03557	fis; Fis family , factor for inversion stimulation protein
PA1097	K10941	FleQ, flagellar regulatory protein
PA4147	K21405	acoR; acetoin dehydrogenase operon transcriptional activator
PA4021	K21405	eatR; regulator of ethanolamine catabolism
PA2665	K12266	norR; anaerobic nitric oxide reductase transcription regulator

PA1050	K02647	yeaG; cdaR; hypothetical protein; carbohydrate diacid regulator
PA0487	K02019	modE; molybdate transport system regulatory protein
PA3864	K21829	dauR; D-arginine utilization repressor
PA0782	K13821	putA; RHH-type , proline utilization regulon repressor / proline dehydrogenase / delta 1-pyrroline-5-carboxylate dehydrogenase.
PA0857	K05527	bolA; BolA family , general stress-responsive regulator
PA5562	K03497	spoOJ; ParB family , chromosome partitioning protein
PA0873	K03721	PhhR; tyrA and aromatic amino acid transport
PA2449	K03721	gcsR; Fis family transcriptional regulator
PA1009	K03567	hypothetical protein;
PA5274	K06140	rnk; regulator of nucleoside diphosphate kinase
PA0403	K02825	pyrR; pyrimidine operon attenuation protein
PA4182	K07734	hypothetical protein; Transcriptional regulator PAI 2-type
PA0405	K07735	hypothetical protein
PA4723	K06204	dksA; DnaK suppressor protein
PA5536	K06204	hypothetical protein
PA4057	K07738	nrdR; transcriptional repressor NrdR
PA4421	K03925	yabB; cell division protein MraZ family
